# A psycho-educational intervention programme for parents with SGA foetuses supported by an adaptive mHealth system: design, proof of concept and usability assessment

**DOI:** 10.1186/s12911-022-02036-9

**Published:** 2022-11-11

**Authors:** Sara Balderas-Díaz, María José Rodríguez-Fórtiz, José Luis Garrido, Mercedes Bellido-González, Gabriel Guerrero-Contreras

**Affiliations:** 1 Department of Computer Science and Engineering, University of Cádiz, Cádiz, Spain; 2 Software Engineering Department, University of Granada, Granada, Spain; 3 Biosanitary Research Institute (Ibs.Granada), Granada, Spain

**Keywords:** Pregnant women, SGA foetus, Proof of concept mHealth, Usability, Adaptation, Data and structure modelling

## Abstract

**Background:**

Technology-based approaches during pregnancy can facilitate the self-reporting of emotional health issues and improve well-being. There is evidence to suggest that stress during pregnancy can affect the foetus and result in restricted growth and preterm birth. Although a number of mobile health (mHealth) approaches are designed to monitor pregnancy and provide information about a specific aspect, no proposal specifically addresses the interventions in parents at risk of having small-for-gestational-age (SGA) or premature babies. Very few studies, however, follow any design and usability guidelines which aim to ensure end-user satisfaction when using these systems.

**Results:**

We have developed an interactive, adaptable mHealth system to support a psycho-educational intervention programme for parents with SGA foetuses. The relevant results include a metamodel to support the task of modelling current or new intervention programmes, an mHealth system model with runtime adaptation to changes in the programme, the design of a usable app (called VivEmbarazo) and an architectural design and prototype implementation. The developed mHealth system has also enabled us to conduct a proof of concept based on the use of the mHealth systems and this includes data analysis and assesses usability and acceptance.

**Conclusions:**

The proof of concept confirms that parents are satisfied and that they are enthusiastic about the mHealth-supported intervention programme. It helps to technically validate the results obtained in the other stages relating to the development of the solution. The data analysis resulting from the proof of concept confirms that the stress experienced by parents who followed the mHealth-supported intervention programme was significantly lower than among those who did not follow it. This implies an improvement in the emotional health not only of the parents but also of their child. In fact, the babies of couples who followed the mHealth-supported programme weigh more than the babies of couples under traditional care. In terms of user acceptance and usability, the analysis confirms that mothers place greater value on the app design, usefulness and ease of use and are generally more satisfied than their partners. Although these results are promising in comparison with more traditional and other more recent technology-based approaches.

**Supplementary Information:**

The online version contains supplementary material available at 10.1186/s12911-022-02036-9.

## Introduction

Information and communication technologies (ICT) provide methods and tools for developing useful systems and are a major step forward in healthcare [1]. Healthcare professionals and patients can benefit from the use of mHealth (mobile Health) systems [2, 3]. These systems enable information to be universally acquired, stored, visualised and processed thanks to the use of mobile software applications (apps) on portable computer-based devices (e.g. smartphones and tablets). This can provide major advantages when monitoring, diagnosing and treating diseases [4] and in the field of active ageing [5]. While mHealth systems currently provide powerful features, it is necessary for them to also possess certain properties such as usability, adaptation, confidentiality, etc. so that they may be successfully accepted and adopted by end-users. This could well represent the first step to improving medical procedures and protocols in terms of efficiency, productivity and resource management for both suppliers and users [6].

Emotional management problems increase the risk of having an SGA and/or premature baby and the number of such cases is currently increasing at an alarming rate [7]. There is evidence to suggest that stress during pregnancy causes alterations in the vascular, neuroendocrine and immune systems of the foetus [8] and increases the likelihood of foetal restricted growth and preterm birth [9]. Machine learning techniques can be used to predict whether the foetus will be born small for its gestational age. In their article [10], the authors propose the *bagged tree* hybrid model which offers an accuracy of 0.849 and can be used to predict such a situation.

A number of studies have demonstrated the relationship between maternal stress and future mild problems in children which become apparent from a very early age with low neurobehavioural performance [11] until adolescence with emotional and cognitive problems [12]. Other severe problems might include lower or impaired mental and motor development, or behaviour disorders such as attention-deficit and hyperactivity disorder [13, 14]. The most common stressors during pregnancy are fears about pregnancy problems, baby malformations, future baby care or changes in lifestyle. These make mothers more vulnerable to major depressive disorder and stress [15]. Stress can also contribute to the onset of depressive problems [16]. Stress and depression should therefore be analysed together within a single model of emotional health in pregnant women and couples.

The application of mHealth systems to pregnancy processes could facilitate the self-reporting of well-being and emotional health (i.e. depression or stress) and provide information to bring about changes and support therapeutic interventions [17]. Prevention, protection and rehabilitation interventions, such as lifestyle changes through longitudinal monitoring during pregnancy, should be implemented in order to improve emotional management and reduce negative consequences [18].

In this paper, we will present our work for designing and conducting a proof of concept for an interactive, adaptable mHealth system. This is intended to support a psycho-educational intervention programme for parents (pregnant women and their partners) with SGA foetuses. The proposal encompasses a number of important areas such as psychological support in emotional management, healthcare, medical advice and communication with the foetus (especially for stimulation purposes).

## Background

The study published in the article [19] focused on the use of mobile apps to monitor pregnant women. Some of the main topics included within the app were to monitor changes in the pregnant woman’s body, provide reassurance, facilitate contact with other pregnant women, etc. However, participants complained about the validity of the content of these apps, and expressed concerns about their security and privacy.

In their article [20], the authors performed a qualitative analysis on two pregnancy apps (Text4Baby and Baby Center’s ‘My Pregnancy Today’) which are used to consult medical information. The study concluded that the second app was more complete and addressed more topics, such as body and management of pregnancy symptoms, foetal development, prenatal care, maternal well-being and partner roles.

In their article [21], the authors analyse how apps consider the role of the father during pregnancy. A number of apps patronise expectant fathers and trivialise their role with the assumption that they need entertainment, humour and encouragement to engage them. The authors suggest focusing on wider social expectations, norms and paradoxes in relation to the role of men in contemporary parenthood. Further research is required to explore how men engage with the apps, and how apps contribute to their understanding and practices as an expectant or new father.

The success of interactive information systems in clinical environments largely depends on their usability so that they can be used by people with limited technological knowledge. A user-friendly system is the one which increases productivity, decreases critical errors, reduces the need for user training (in both clinical staff and patients) and improves user acceptance. In order to be used in clinical research, an mHealth system requires a more rigorous usability evaluation due to the limitations of mobile devices and applications [22].

The Health IT Usability Evaluation Model (Health-ITUEM) [23] is based on the technology acceptance model (TAM), ISO 9241-11 (efficiency, effectiveness, learnability and satisfaction) and Nielsen’s [24] and Shneiderman’s [25] design guidelines. The Health-ITUEM model evaluates nine concepts which are classified into two categories: the perceived usefulness (flexibility/customisability, learnability, performance speed, competency and completeness); and the perceived ease of use (error prevention, memorability, information needs and other outcomes), and impact and user control. This model was tested in two experiments with the participation of 60 adolescents in focus groups. In the first experiment, 32 adolescents answered four questions about mobile technology: how they used it, why they used it, what barriers they encountered when using it and what strategies they used to overcome such barriers. In the second experiment, 38 adolescents also participated in a focus group after they had used four different health apps for a month. They answered the same questions as in the first experiment with the exception of the first. The free text responses to the questions in the focus groups were analysed and coded as positive, neutral or negative. Inter-rater reliability was calculated with a greater than 90% agreement for every code except Flexibility/Customizability which was calculated at 56%. The usefulness of the model was demonstrated. Some of the limitations of the model are that only adolescents participated in the study, it is a qualitative method with focus groups that require moderation, and participation and responses need to be analysed so that the information can be extracted and coded. In a subsequent, more recent study, the sample consists of 92 adults living with HIV and they used and evaluated the usability of a new mobile app for symptom self-management [26]. In order to validate its usability, participants completed the Health-ITUES and the Post-Study System Usability Questionnaire (PSSUQ). Correlations between these scales and each of the subscales were assessed particularly in terms of perceived usefulness and impact factors.

In their article [27], the authors analyse Bloom, a pregnancy application. Its objectives include minimising risk factors, motivating healthy behaviour and encouraging self-monitoring by providing daily goals which are adapted through the course of pregnancy, offering credible information and improving communication between medical staff and expectant mothers to reassure the mother. The application was co-designed with the participation of pregnant women. Intervention focused on tracking and providing customised feedback (i.e. a daily dashboard) on nutrition, hydration, activity, weight, symptoms and mood. Its usability was evaluated by five participants using semi-structured interviews and the system usability scale (SUS) questionnaire [28]. The answers enabled improvements to be made to the information architecture and the task flow. The participants suggested a number of usability improvements and these include customizing the goals to accomplish with their prenatal care staff and visualising nutritional information according to pregnancy-relevant criteria such as vitamin levels, macronutrients and food groups. The “insight” dialogues provided by the tool were also redefined since this information caused the participants to become increasingly anxious and so these now appear in the form of recommendations and explanations.

The SUS questionnaire was also used by 20 participants to test the final mobile application created in the article [29] and this was designed following a user-centred approach to help midwives support antenatal care (ANC) in primary healthcare. Antenatal care includes assessing body weight, measuring blood pressure, upper arm circumference, fundal height and foetal heart rate, recording tetanus immunisation status, administering or prescribing iron tablets, conducting laboratory tests, providing training and managing each case. Analysis of the test results reveals it to be an effective tool although there were two drawbacks: users require the help of a technical person to use the system and they also need to learn many things beforehand.

In their article [17], the authors conduct a 6-week experiment with 38 participants (pregnant and non-pregnant women and clinicians) to co-design a clinical interface for an app to monitor pregnancy and well-being. The app should share a broad spectrum of data with clinicians, connect such data to goals and values and prioritise the midwife-patient relationship. Once the prototype had been designed and implemented, the authors performed a qualitative evaluation by means of personal interviews. The main concern was to guarantee privacy and security when the health data is shared, particularly where mental health data is concerned. The same problem was identified in another article [30] which conducted a study of midwives working at an Australian hospital. Their attitudes and experience in the use of ICT were analysed to identify potential causal factors that might encourage or inhibit their use in prenatal care. Evaluation was performed through semi-structured interviews (8 participants) and focus group sessions with short surveys (13 participants). The results showed that although the midwives recognized the potential benefits of using ICT, they also expressed reservations about the lack of training, skills and motivation and the perceived legal risks associated with social media applications.

A more complete study [31] reviews and categorizes 47 mobile apps. Most of these include information about the signs of risk and disease during pregnancy, physical changes relating to a regular pregnancy and prenatal education, but they also highlight the fact that users are concerned about the reliability of sources and expert opinions.

In their article [32], the authors evaluated usability in terms of feasibility and acceptability and assessed the effectiveness of the mHealth lifestyle and medical apps to support healthcare during pregnancy in high-income countries. They review 19 apps with different objectives: to reduce gestational weight gain by increasing fruit and vegetable consumption, to stop smoking and to support healthcare for the prevention of asthma and infections during pregnancy. Evidence on the usability and effectiveness of these apps is limited since in most cases the adopted assessment methodology is unclear. The authors therefore conclude that further research into usability is required before such healthcare applications are implemented.

In another article [33], the authors provide 39 specific guidelines for designing mobile devices. The guidelines were obtained from a review of literature to identify usability experiments on mobile applications and websites. These guidelines are grouped into five categories: layout, navigation, design, content and performance. Although these offer an important contribution, they need to be updated and are only intended to be used by experts and designers and have not been created specifically for eHealth systems.

Design guidelines for usable eHealth apps are proposed in another article [34]. Although these only relate to navigation and design, they are more specific: they use user representations (avatars and emoticons), they avoid the use of the scroll bars and redundant clicks, they use displayable menus and they limit the layout and number of screens to four (one for authentication, one for the welcome, one for the blog with information and one for the chat with spoken turn-taking).

In summary, there are a number of proposals based on mHeath systems and apps for monitoring pregnancy. While most studies designed mHealth intervention programmes for healthy pregnancies [35]. None, however, has been created to specifically address the case of parents at risk of having an SGA or a premature baby through a supervised psycho-educational intervention programme. In terms of usability and design, very few studies follow modern and current design guidelines for apps. Our proposal will jointly consider several of the aspects usually addressed separately by such work. We will therefore address the design and assessment of the mHealth system in terms of the app UI (design, navigation, notification and functionality) in order to support a complete psycho-educational programme (medical advice, healthcare, foetus stimulation and parents’ emotional management), and also to guarantee properties such as data security and privacy.

## Methods

This section describes the methodology followed to develop our approach, and also the participants, instruments and procedures used to conduct the proof of concept and to assess acceptance and usability. We will also describe previously introduced methods [36] in further detail.

### Methodology

**Fig. 1:**
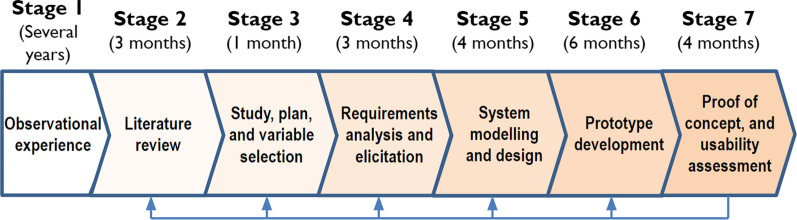
Stages of the methodology

We have adopted a software development-based methodology [37] which comprises seven main stages (Fig. 1) and in each stage, certain aspects from previous stages are revised, modified and completed. Our methodology therefore follows the main objective of our proposal, i.e. to provide suitable technological support (e.g. an adaptive and usable mHealth system) for an intervention programme. When technology is particularly important in research development so that users can perform their tasks, the adoption of this kind of methodology can be critical at each development stage since it is necessary to ensure that end-user needs are satisfied. For instance, in their article [38], the authors adopt an adapted software development life cycle model in order to design, develop and test an app which is intended to support bedside nursing care, and in another article [39], the authors studied the order of analysis-design-implementation-evaluation (according to the standard lifecycle of system development) for the development of an app to manage clinical-guideline-based obesity. The common and main phases in this kind of methodology but adapted to our research work are described below.

The starting point for this research first arose from an early phase lasting several years whereby out team (midwives, doctors, psychologists, etc.) observed the daily clinical practice of healthcare pregnant women. We frequently assist women with SGA foetuses in pregnancy control sessions, and as the parents’ emotional state will also impact on their daily lives, they thought it would be beneficial to provide a psycho-educational intervention programme which could be managed by mothers and their partners in a continuous, structured and ecological way.

A review of the literature was carried out in the second, third and fourth stages. The literature review allowed the healthcare professionals, psychologists and software engineers in our team to study and prepare an initial plan of action in the third stage. This plan of action was based on goals and took into account the variables that were selected to devise the technological solution to support the programme.

In the fourth stage, technical and functional requirements and properties were elicited from the goals by applying requirements analysis techniques such as expert interviews and focus groups to gather quality information about those involved and the organisational aspects, and brainstorming to gather new ideas, opportunities and improvements. This enabled the engineers in our team to identify the hardware and software technology necessary to provide the required support for the programme. In particular, the hardware requirements included the need for a server computer, the use of end users’ mobile devices and Internet access for the connection between the server and mobile devices, and resulted in a decision being taken based on the BYOD paradigm (or bring your own device) [40] whereby powerful and advanced mHealth solutions can be developed on account of the fact that every user has their own smartphone with Internet connection. The main properties gathered for consideration on a technological level included adaptation, usability, reliability and security. In order to satisfy these properties, specific design decisions were taken in the subsequent stage. A more detailed explanation of these properties and functionalities can be found in another article [36].

In the fifth stage, the system was modelled and designed using task analysis and modelling. Task analysis and modelling methods have traditionally been used to provide a rigorous, structured description of the end-user activity required for system design [41]. By applying this method, and from the functional requirements elicited in the fourth stage (e.g. to provide guided navigation for mothers and partners, show progress in the programme to healthcare professionals, etc.), it was possible to organise the contents of the intervention programme into a task model with different types of resources (explanations, instructions, activities, questionnaires, etc.). This stage also resulted in the architectural design of an mHealth system comprising the front-end subsystem with an app (called VivEmbarazo) for use on the end user’s mobile device, and a back-end subsystem with an author tool, database management system and adaptation motor, all of which are deployed on a server (back-end subsystem).

The aim of the sixth stage was to develop a fully operational prototype of the mHealth system based on the resulting design of the previous stage. During this stage, the prototype was implemented and tested in the laboratory by the software engineers with the active collaboration of the healthcare professionals on the team.

In the seventh and final stage, we conducted a proof of concept and assessed acceptance and usability with the participation of a group of parents who were at risk of having an SGA baby and who used the prototype for approximately 16 weeks. The proof of concept and its assessment are described in the following subsection.

### Proof of concept

The proof of concept in the seventh stage of the methodology is based on the variable selection performed in the third stage. The selection of variables was actually critical for other stages in the methodology and this will be detailed later in this article. The selection of socio-demographic, biomedical and emotional health variables is shown in Table 1.

**Table 1:** Socio-demographic, pregnancy and emotional health variables

Variables	Measures and tests
Socio-demographic	Ages of mother and partner
	Cohabitation of the couple
	Time of relationship of the couple
	Mother’s educational level
	Partner’s educational level
	Mother’s job
	Partner’s job
	Sex of the baby
Pregnancy	Number of pregnancies
	Number of abortions
	Number of deliveries
	Gestational week
	Birth weight
	Obstetric risks (natural, operative vaginal, caesarean)
	Biomedical risks (high blood pressure, obesity, diabetes)
	Social risks (mother over 35 years old, smoker, consumer of alcohol, drugs)
Emotional health	Perceived stress scale (PSS) [42]
	Spanish-language version [43]. This scale measures the extent to which life situations are considered to be stressful. The scale ranges from 0 to 40 (with higher scores representing higher stress levels)
	Edinburgh Postnatal Depression Scale (EPDS) [44, 45]. This scale assesses the mother’s mood. Cut-off values of 10 or higher are most often used to identify women who might suffer from depression

The proof of concept is based on an experimental study with a control group. The selection criteria for the participants was a pregnancy diagnosis with a risk of an SGA baby. The parents chose a ball from a bag to determine whether they would be assigned to the experimental or to the control group. A total number of 96 people participated in the experiment with 24 couples assigned to the experimental group and 24 couples to the control group. A sample analysis has been carried out during 2019 and 2020 and this will be presented in the results section.

We used other additional instruments for this proof of concept in addition to traditional ones such as initial tests to report on the state of health and to obtain demographic values collected at individual appointments. We analysed the data obtained and this is presented in the results section.

In terms of procedure, the researchers presented the study to the participants at a first meeting. The couples then ran the app by entering a previously assigned user code so that their answers could be collected. The participants also had to complete a number of questionnaires associated with the tasks available in the app.

The study was approved by the Ethics Committee of the University of Granada and informed consents were obtained from the participants to be included in the study and the proof of concept then began. The participants of the two groups also completed various clinical tests to assess both their physical and emotional state of health (e.g. PSS and EDSS included in Table 1). Every activity was supervised by the healthcare professionals. The information was also completed by the doctors with the results from the clinical sessions.

The experimental group participants followed the programme by using the *VivEmbarazo* app for 16 weeks (second trimester and part of the third trimester of the pregnancy). The app allowed them to learn about specific topics, to carry out the proposed activities to strengthen training and to complete a number of questionnaires to assess learning and app usability and acceptance (see Figs. 6 and 7 , and Additional file 1: Tables 1 and 2). At the same time, the control group participants were told about the same topics and the activities to be performed using traditional procedures (i.e. without using the mHealth system).

Two specific questionnaires were added to assess app usability and acceptance (see Additional file 1). The questions were based on certain guidelines used for app design (as we will mention in the App User Interface subsection) and on previous questionnaires used in the literature reviewed [23, 26, 28]: SUS, PSSUQ, and TAM tests in addition to the concepts assessed in Health-ITUEM. In order not to overwhelm parents at the end of the programme, questions about usability and acceptance were included on different questionnaires when the parents were halfway through the test and until the end of the intervention programme and once they had already undertaken a number of sessions.

## Results

In this section, we will present the most relevant results split into multiple categories according to the different stages of the approach. The first, four relevant results are as follows: the metamodel which supports the task modelling of current or new intervention programmes; the mHealth system model which includes key components to enable runtime modifications to be made to the programme; the design of a usable user interface (UI) for the app; and the architecture and prototype implementation. By way of conclusion, the proof of concept includes data analysis and usability assessment to confirm end-user satisfaction and acceptance of the mHealth system for carrying out the intervention programme. This also enables us to validate the results obtained in the other stages relating to the development of the technological solution.

### Task model

Based on functional requirements, the focus of the mHealth system design was on the process (or workflow) that parents must follow periodically (ideally daily) in order to carry out the intervention programme. The application of the task analysis and modelling methods results in a specific task model, and this enables us to structure all the contents accessed by parents participating in the programme. It also allows us to base the design of the mHealth system and its app on the user’s tasks and the relationships between these.

**Fig. 2:**
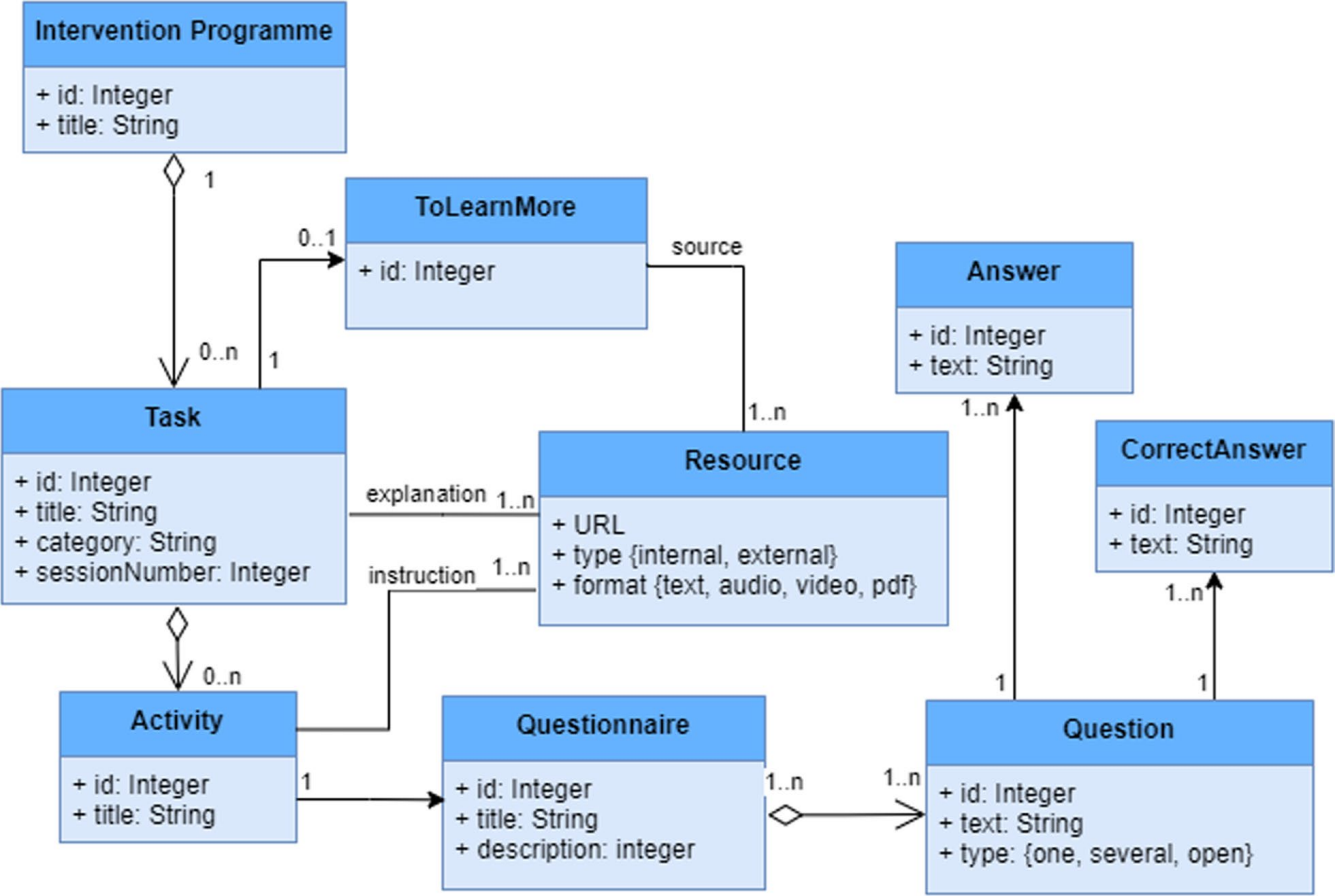
Metamodel for building the task models

The main result of this research is the creation of the model shown in Fig. 2 which serves as a metamodel for building the task model for any other programme under the philosophy at the heart of our approach. This metamodel is also required for implementing the adaptation mechanisms which support the runtime programme modifications and the author tool and these are described in the following subsection.

The metamodel in Fig. 2 can be described as follows: each task includes various sections such as a general explanation presented with multimedia resources and zero or more activities to be carried out. Certain tasks also include an additional “To learn more” section with links to documents (and their source) with verified specialist information. Some of the mother’s activities and instructions for certain tasks might vary from those of her partner.

Each activity comprises a series of instructions which are presented as resources in different formats to explain what needs to be done and how, and one or more questionnaires. In order to perform certain activities, additional files containing multimedia content provide the required resources (e.g. a baby-stimulation song) and sometimes the activities may need to be performed in specific places/conditions (e.g. while the mother is lying on the bed).

The questionnaires collect information about activity progress and accomplishment, and comprise one or more questions. There are three types of questions depending on the number and kind of answer: choose one answer in a list, choose several answers in a list or write an open answer. The first type is used when several Likert values are presented (e.g. “Do you think this activity is useful for communicating with your baby? Score this on a scale from 1 to 5 where 1 = Not at all and 5 = Very much”). Additionally, certain questions have a definite correct answer to automatically check whether the user’s answer is correct.

### System model

**Fig. 3:**
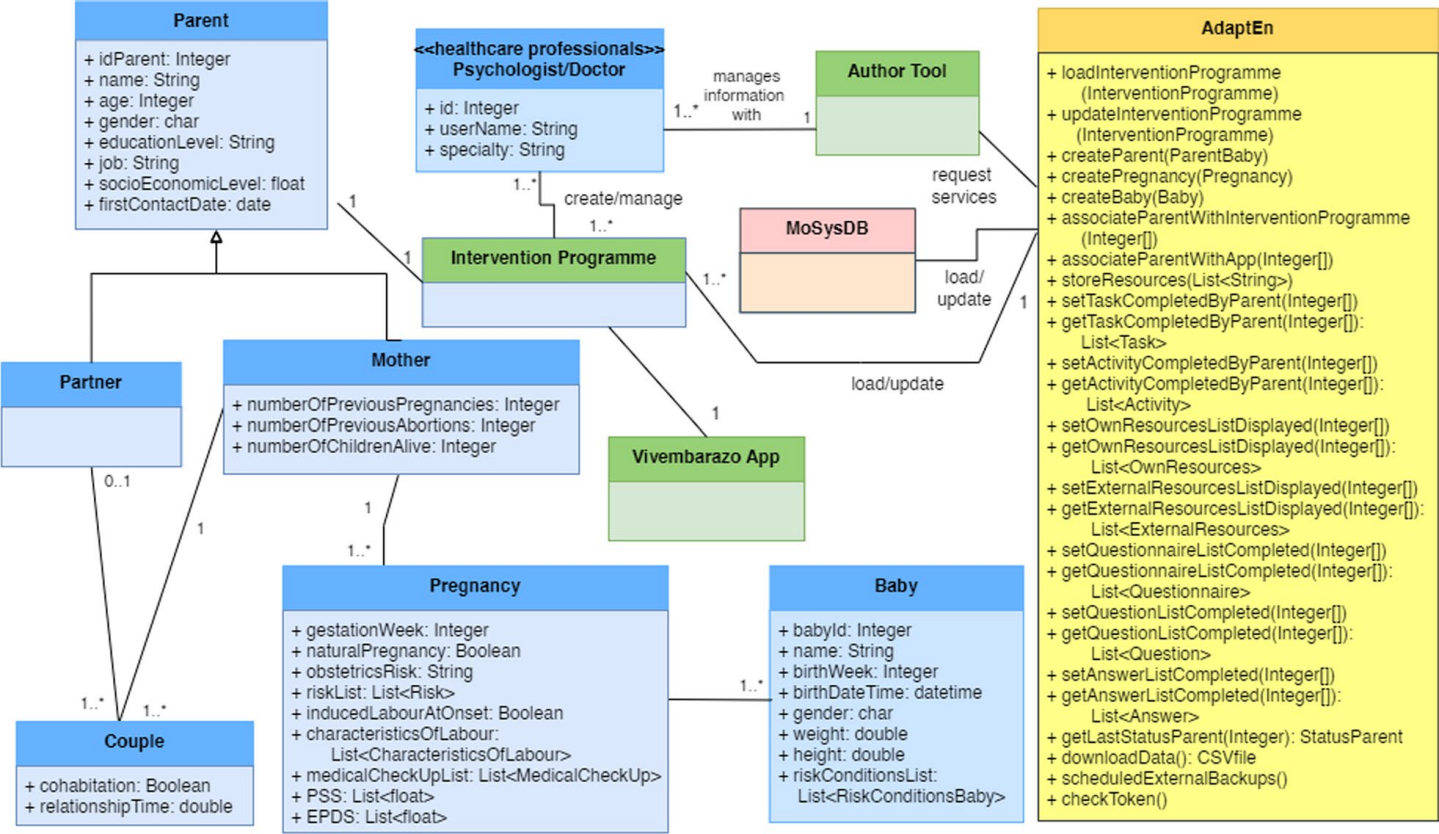
System model

The domain model for the mHealth system is another model that is useful for the design stage. This model is also built on the basis of the previous requirements analysis and task model. It comprises the following types of entities (Fig. 3):

- *People* these include parents (mother and her partner, with both forming a couple) and the baby or foetus, in addition to the professionals involved in programme design and management (all marked in blue).
- *Applications* these refer to the *VivEmbarazo* app used by parents to follow the programme and the web-based author tool to configure and modify an existing programme or to design a new one (both marked in green).
- *Run-time adaptation module* the adaptation engine (*AdaptEn*) module is responsible for implementing the mechanisms supporting the adaptation at run-time. It follows a component-based design whereby different services are invoked (marked in yellow).
- *Database* this is the database management system (*MoSysDB*) to support the information system that stores all the data about the participants and their progress on the programme in terms of completed tasks, questionnaire answers, etc. (marked in pink). We use MariaDB technology because it is open source and has been extensively tested.

### Adaptation

The *AdaptEn* module enables the mHealth system to be adapted as follows:

1. New intervention programmes can be added and the content of existing ones can be modified and associated with existing or new parents with babies.
2. The programme for each mother and partner can be individually changed when it is already in progress, for instance when the healthcare professionals consider other different or additional tasks/activities to those initially planned.
3. The programme can be created in different languages and includes multimedia resources in these languages.

Each mother or partner can also follow a different intervention programme that can be adapted to their needs. Professionals use the author tool to define or adapt these programmes and the *AdaptEn* module will then be responsible for automatically compiling the app from this content for a specific user whenever the *VivEmbarazo* app is run.

The *AdaptEn* module supports the following adaptations through various services in coordination with other model entities (Fig. 3):

1. Automatic *loading* and *updating* of programmes in the system, and the association of these to parents
2. *Inclusion* of the necessary elements in each specific programme (e.g. tasks, activities, resources, questionnaires, etc.)
3. *Monitoring* the degree to which parents have carried out and completed the programme
4. *Downloading* and *backing up* user interaction data
5. *Security* of the stored and transferred data

The programme is loaded (*loadInterventionProgramme* service) and updated (*updatingInterventionProgramme* service) when the healthcare professionals interact with the author tool to create and modify a programme. The programme can also be associated with parents who are added to the system as necessary (*createParent* and *associateParentsWithApp* services). There is a connection to the database *MoSysDB* to store all the programme resources (*storeResources* service), including multimedia files (videos and audios) and links included in the app.

*AdaptEn* accesses *MoSysDB* to manage all the information collected from user interaction with the app, including the tasks and activities performed, the content and resources that have been accessed and the questionnaire answers (*set* and *get* services). *AdaptEn* also saves the last state of execution of the app by each parent (*getLastStatusParent* service) so that they can continue the programme the next time at exactly the same point from where they had stopped. Two services are also included to obtain or preserve the data: the *downloadData* service to download the questionnaire answers provided by the parents and the *scheduledExternalBackups* service to schedule backups of key data from the same database to external servers.

Finally, backups, user information privacy and security are covered by the *checkToken* service which establishes a secure connection and encrypts the information transmitted over the network.

### App user interface

Technology that is not user-friendly will decrease user productivity and interest and reduce app usefulness, thereby resulting in a reduction in the number of users. Well-known usability guidelines can be followed during design so that applications are usable. In our case, parents will use the app for several minutes each day. Certain design guidelines [46] have been observed not only to guarantee usability but also to ensure that different models are compatible. User interface (UI) patterns and icons from Material Design by Google (https://material.io/design) can also be used to ensure user familiarity.

Another result from the fifth stage regarding app design is summarised in Table 2 and these include the main guidelines associated with different usability aspects considered such as UI design and navigation, notification management and the presentation of the functionality in addition to aspects relating to security. The designers also considered these guidelines when testing the app.

Additional usability guidelines were also considered such as the guidelines for designing graphical UI of mobile E-Health [34]. The authors propose a responsive UI layout, the use of no more than four screens (authentication, task menu, task section menu and task section) and the inclusion of a scrollbar and displayable menus with clear and easy navigation to avoid redundant clicks. In our case, the app does not require a chat and so the guidelines relating to this feature do not apply.

**Table 2:** Usability guidelines for the *VivEmbarazo* App

UI design	Consistency: user interface elements should be consistent both in terms of look and behaviour
	Layouts should be responsive
	Information should be presented in a single, standard column with one-directional scrolling
	Buttons and sections in tasks should stand out and be finger friendly
	Language and text used in the “To learn more” section should be clear, concise, and syntactically and orthographically correct
	Texts can be enlarged
	Suitable, contrasting colours should be used
	Colours are used to differentiate changes of state, such as task completion
Navigation	Navigation should be guided by the order of the tasks, activities and questions, and these appear one at a time so that they can be completed successfully
	Hierarchical navigation should be clear: tasks—sections—content A minimum number of steps should be required to reach a specific functionality (in our case, 3)
Notifications	Error messages should be clear
	Permission for notifications is required to suggest that users complete new or pending sessions
	Advertising and content unrelated to the core information should not be shown
Functionality	Multimedia content for each section in a task should open correctly
	Audio should stop when the screen is off
	A single click should be needed for interaction
	No time limit should be set to read and view content and to complete questionnaires
	Graphics, text, images and other UI elements displayed in the app should not be distorted, blurred or pixelated
	No services should be running when the app is in the background
	When the app returns to the foreground, the app should return the user to the same place as when last used
Security	User identification should guarantee security for app access
	Data should be encrypted in the database and during transfer
	All network traffic should be sent over SSL, HTTPS and a Network Security
	Configuration file should be defined to customise security
	Access tokens should be requested to ensure that users are authenticated
	Content should not be shared with other applications or systems
	The system should comply with data protection regulation

**Fig. 4:**
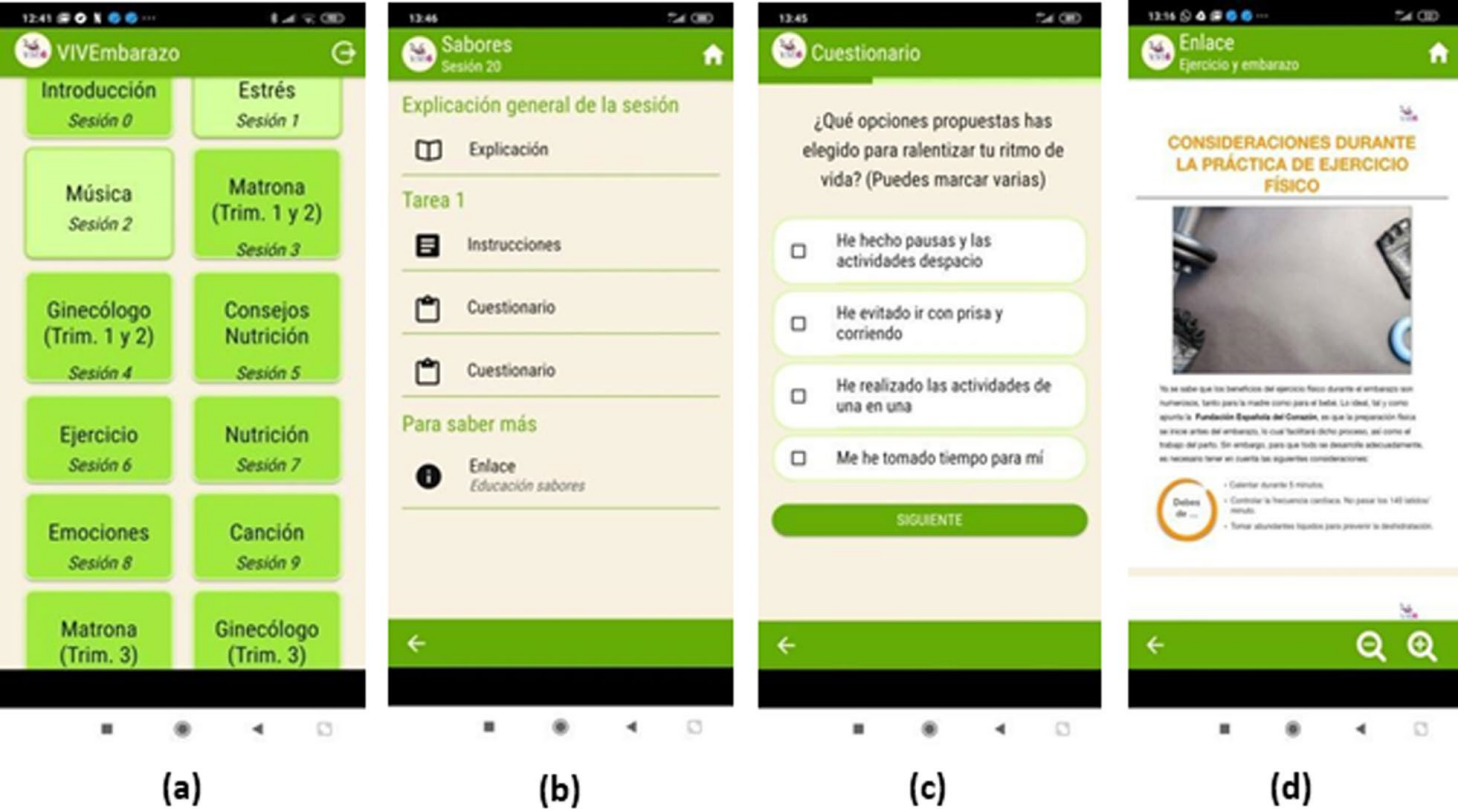
**a** Menu of tasks, **b** sections in a task, **c** questionnaire, and **d** “To learn more”

Figure 4 shows the UI created for the app corresponding to the task model built as an instantiation of the metamodel shown in Fig. 2. For this specific psycho-educational intervention programme, this follows the guidelines outlined in Table 2. Figure 4 shows: 4a a menu of tasks ordered according to session; 4b a task with several sections, i.e. explanation, activity with instructions and two questionnaires, and a “*To learn more section*” with a link to a resource; 4c a question from a questionnaire; and 4d a document in a specific “*To learn more*” section within a task. Most of the task contents are multimedia resources.

The example in Fig. 4 corresponds to the prototype developed for our specific intervention programme. This consists of one introduction task to present the application and its objectives, and 20 additional tasks (Part 4a in the figure). In this programme, the tasks are classified into four main pregnancy areas: medical advice, healthcare, communication with the foetus for stimulation purposes and emotional management. For instance, the area of medical advice includes the following tasks: *3 Midwife* (Trimesters 1 and 2), *4 Gynaecologist* (Trimesters 1 and 2), *10 Midwife* (Trimester 3), etc. The numbers in the tasks indicate the preferable order that the daily sessions should be conducted and this is independent of the area to which the tasks belong. Part 4b in the figure shows an example of a task with three sections: General Explanation, Activity 1 and “To learn more”. The Activity 1 section includes Instructions and two questionnaires. The To learn more section includes a link to a resource in pdf format. Part 4c in the figure shows a multiple-choice question from a questionnaire with checkboxes for the possible answers. The final part of the Fig. 4d shows the pdf file which is opened when the user selects the To learn more section. Although the figure shows screenshots of an intervention programme in Spanish, healthcare professionals can create content in other languages.

### Architecture for the prototype implementation

Nuzzo et al. [47] discuss the usefulness of technological methods and tools (the design and architecture of a software system) which enable phenotypic information to be flexibly managed. In a similar way, the previous task model, the system model and the architecture described in this article enable the interactive, adaptive mHealth prototype to be developed. This consists mainly of an app executed by end-users on their mobile devices (frontend) and a database management system and an adaptation engine which are both executed on a server (backend).

**Fig. 5:**
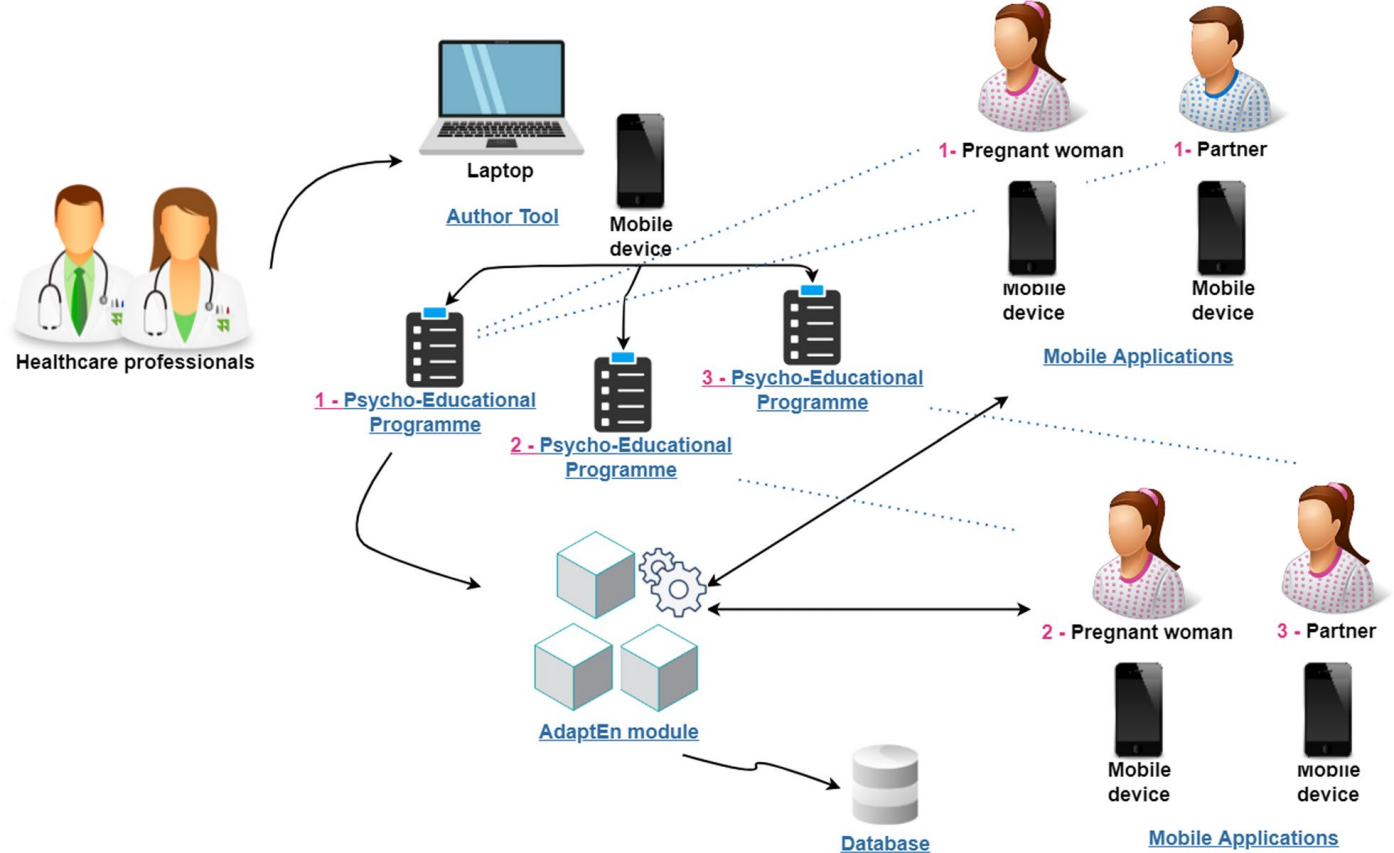
Architecture

Figure 5 presents the architecture comprising those involved, the intervention programmes, the *AdaptEn* module, the *MoSysDB* database, the author tool, and the mobile applications and devices. It shows that there are three different intervention programmes (i.e. 1—Psycho-Educational Programme, 2—Psycho-Educational Programme, and 3—Psycho-Educational Programme) that can address different aspects according to specific user needs while they are under constant supervision by professionals of healthcare. New psycho-educational programmes can be added or modified at runtime using the *AdaptEn* module which connects with *MoSysDB*.

The proposal follows a multi-layered architectural model. The presentation layer hosts the interface, and the presentation logic for the *VivEmbarazo* app and the author tool. The author tool is deployed on an external server and is accessible using standard web technologies (i.e. browsers). The *AdaptEn* module is hosted on a cross-cutting services layer. Clients connect to the server (the app and the author tool), make a request (HttpRequest) and the response message is encapsulated in an HTTP-response. Finally, the database is also deployed on the server.

The *MoSysDB* database scheme is generic so that it is able to host different intervention programmes for various health domains and is not limited to that of small-for-gestational-age pregnancies or babies. *MoSysDB* enables the development of monitoring systems and database-driven mobile applications, making it possible to change the internal content of an already installed application without the need for an update or reinstallation. *MoSysDB* also supports the representation of various types of content and formats required in the health domain. This allows engineers to anticipate the future storage needs of other types of previously unknown content in the early design and development stages.

### Data analysis

In a previous article [36], we discussed parental engagement and the degree of their involvement in the tasks in the four main programme areas of the programme. On this occasion, the results presented arise from the proof of concept for which the mHealth prototype was used.

The sample analysis is shown in Table 3. There is no significant difference between the ages of the participants in the experimental group and the control group, or between the mothers and their partners. Nor were there any significant differences in terms of education, employment and social risks for the mothers and partners. Therefore, both groups are similar socio-demographically.

In terms of pregnancy problems, there was no difference between the biomedical risks of mothers in the experimental group and the control group. No differences were found between the experimental group and the control group with respect to emotional health (stress and depression) measured at the detection of the pregnancy at risk (T1).

**Table 3:** Socio-demographic, biomedical, and emotional health of couples who started the mHealth-supported intervention programme

	Experimental group (mHealth programme) N = 24 couples	Control group (Usual care) N = 24 couples	*p*
*Age*			
Mother	31 (27.5–36.5)	34 (30.5–36.5)	0.247
Partner	27 (30–34.5)	31 (36–39.5)	0.106
*Mother’s educational level*			
Primary school	7 (28%)	8 (34%)	0.638
High school	5 (20%)	3 (13%)	
College/university	12 (52%)	13 (53%)	
*Partner’s educational level*			
Primary school	5 (20%)	5 (20%)	0.670
High school	8 (33%)	11 (47%)	
College/university	11 (47%)	8 (33%)	
*Mother’s job*			
Employed	14 (60%)	16 (67%)	0.680
Unemployed	10 (40%)	8 (33%)	
*Partner’s job*			
Employed	21 (87%)	16 (67%)	0.135
Unemployed	3 (13%)	8 (33%)	
*Social risks*			
Smoker	2 (8%)	3 (12%)	0.640
Mother over 35 years old	3 (12%)	3 (12%)	
Mother consumer of alcohol, drugs	0 (0%)	1 (4%)	
*Biomedical risks of mothers*			
High blood pressure	17 (69%)	13 (54%)	0.696
Diabetes	2 (8%)	4 (15%)	
Obesity	5 (23%)	7 (31%)	
Mother stress (T1)	25 (22.75–26.75%)	25 (22.50–35.50%)	0.311
Partner stress (T1)	24 (22.50–25.50%)	24 (23–27.50%)	0.614
Mother depression (T1)	12 (10.50–13.50%)	14.50 (12–15%)	0.681
Partner depression (T1)	8.50 (6.50–10.25%)	12.50 (9–13%)	0.876

Data are presented as n(%), chi-square test for type of delivery, studies and work of the mother and partner. Median (interquartile range), Mann Whitney U test for baby birth weight, gestational age at birth, and age of the mother and partner. Social risks have not been taken into account in this analysis

Table 4 shows the preliminary results in terms of the differences observed between the fifteen couples (62.5% of 24 couples) who completed 60% of the mHealth-supported programme, and their randomly assigned controls, receiving the usual hospital care. The differences regarding the obstetrics risks, sex of the baby, birth outcomes, maternal and paternal stress and depression measured 16 weeks after the start of the programme (T2) were analysed. There were 9 couples from the experimental group who did not finish the programme for various reasons: three foetuses died in the uterus, four couples moved to another city and hospital, and two couples dropped out of the study. No differences were found in the socio-demographic variables between those who started and finished the study, nor for mothers ($p=0.197$) or for fathers ($p=0.223$).

**Table 4:** Differences in the obstetrics risks, sex of the baby, birth outcomes, stress and depression of mothers and partners who finished the mHealth-supported programme

	Experimental group (mHealth programme) N = 15 couples	Control group (Usual care) N = 24 couples	*p*
*Obstetric risks of mothers*			
Spontaneous	9 (60%)	4 (15%)	0.017*
Operative vaginal	3 (20%)	7 (31%)	
Caesarean	3 (20%)	13 (54%)	
*Sex of the baby*			
Male	7 (46.7%)	8 (53.3%)	0.695
Female	8 (53.3%)	7 (46.7%)	
*Birth outcomes*			
Baby weight at birth	2.560 (2.450–2.850)	2.070 (1.810–2.565)	0.043*
Gestational age at birth	38 (37–39)	37 (34.5–38)	0.044*
Preterm	2 (13.3%)	7 (46.7%)	0.026*
Full term	13 (86.7%)	8 (53.3%)	
Small gestational age (SGA)	13 (86.7%)	15 (100%)	0.132
Appropriate for gestational age (AGA)	2 (13.3%)	0 (0%)	
Mother stress (T2)	18.50 (14.75–23)	25 (23–31)	0.000**
Partner stress (T2)	15 (15–19.75)	19(16.50–20)	0.538
Mother depression (T2)	11 (8–14.75)	16.50 (15–17)	0.534
Partner depression (T2)	7.5 (7–8)	7(5–9)	0.857

Data are presented as n (%), chi-square test for type of delivery, studies and profession of the mother and partner. Median (interquartile range), Mann Whitney U test for weight and stress of the mother and partner. Stress is measured with the PSS scale (0–40) high score representing high stress level. Depression is measured with the EPDS scale (0–30). Cut-off values of 10 or more are indicative of depression

*$$p<0.05$$, **$$p<0.01$$

The results for babies whose parents followed the mHealth-supported intervention programme showed that birth weight was higher than that of babies whose parents did not follow the programme. The number of preterm babies was also lower and the gestational age was higher in the pregnancies of mothers who followed the programme.

The stress levels of mothers who followed the programme was lower than those of mothers who did not follow it. No differences were observed, however, in partners and this may be due to the fact that they participated less in the programme than mothers.

### App usability and acceptance assessment

**Fig. 6:**
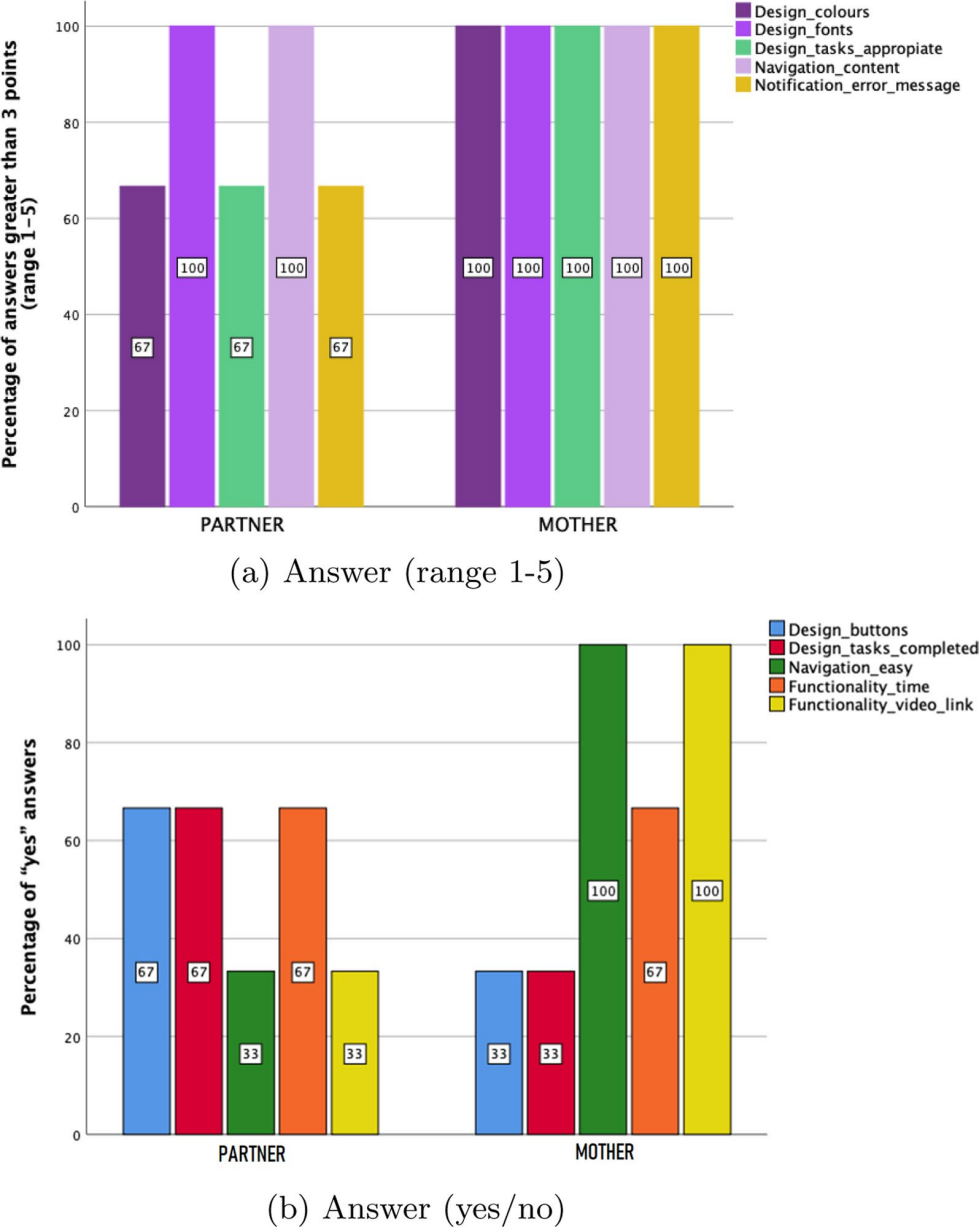
Assessment of the UI design, navigation, and notification by mothers and partners

**Fig. 7:**
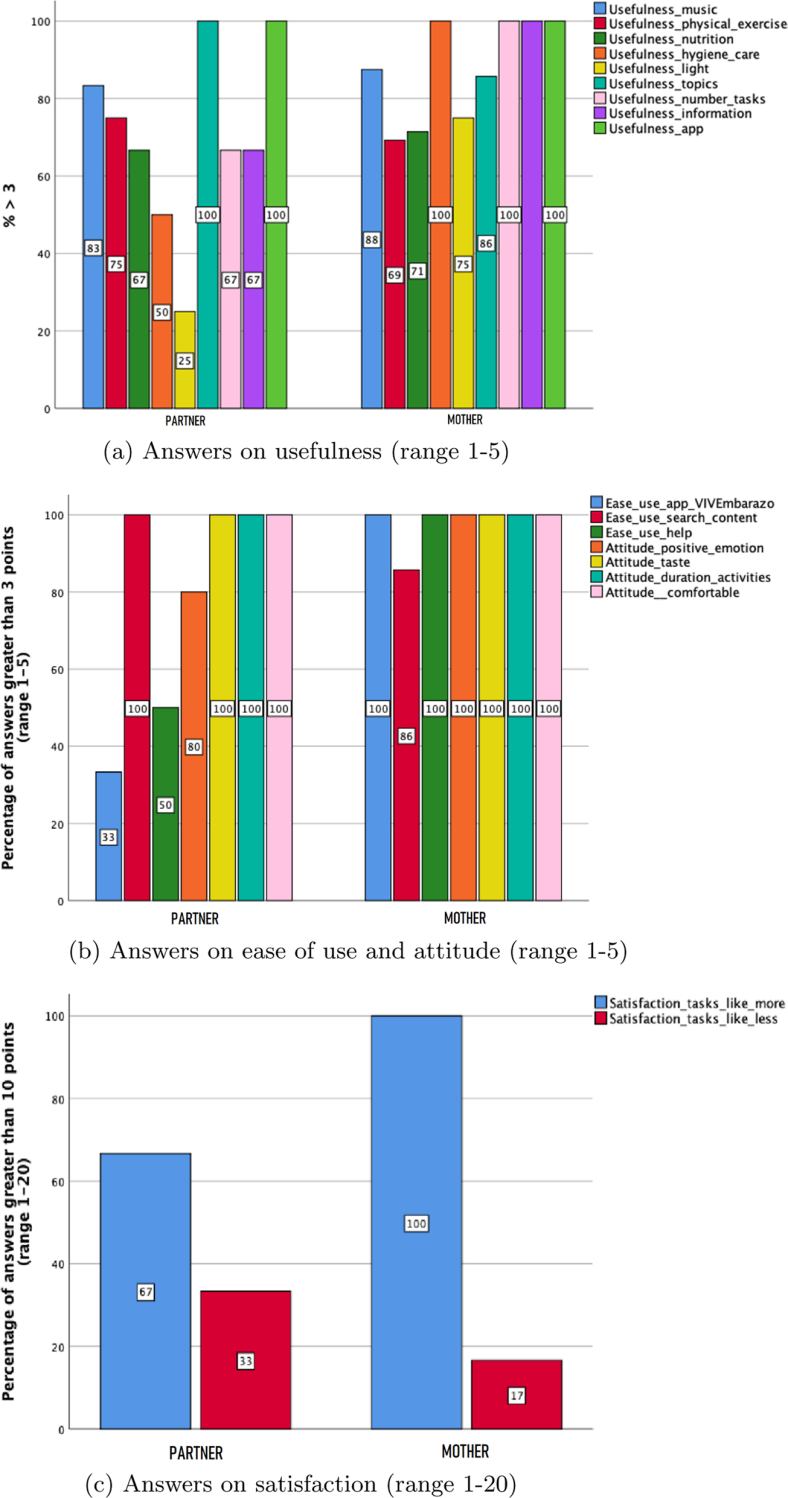
Assessment of technology acceptance by mothers and partners

This subsection presents the results relating to the usability and acceptance assessment from the data obtained from the specific questionnaires completed by mothers and partners who participated in the proof of concept of the *VivEmbarazo* app for the sixteen weeks of the study. The following aspects are assessed: app usability and acceptance (user interface design, navigation, notification, functionality) (Fig. 6); and technology acceptance (perceived usefulness, perceived ease of use, attitude, perceived satisfaction) (Fig. 7). A “good” criterion has been contemplated for app usability and acceptance so that for scores on the Likert scale (1-5), a score higher than 3 is considered “good”. In ordinal scores (No; No, I have only read partially the content; Yes), it is considered “good” to choose “yes”.

Figure 6a shows that the mothers rate the 70% of the design, notifications, navigation and functionality elements as “good” (score>3 or *yes* as an answer). Only two elements of the design (button design and task completion indicators) reach less than 40% of positive responses. Meanwhile, partners rate colours and fonts with the highest score, the 60% of the elements get 67% positive ratings, and only two elements reach less than 40% of positive responses.

Mothers find it easier to navigate the content of the app because they rate this element with 100% positive responses compared to 67% of their partners. Regarding functionality, the results are similar in the time they need to read the messages, but they differ in the functionality of videos and links, 100% positive responses from mothers compared to 33% from partners (see Fig. 6b).

It is observed that mothers and their partners are equally positive about the usability for music, physical exercise, nutrition, topics and the usefulness of the *VivEmbarazo* app. Partners are, however, more negative about the usefulness of hygiene care, of hygiene care, light, number of tasks, and information provided in the “To learn more” (see Fig. 7a). Although partners find it easier to search for specific content of interest in the app, mothers find the app easier to use and do not require help (see Fig. 7b). The mothers and their partners show similar attitudes, except for positive emotions after doing the activities as the mothers say they feel more positive (Fig. 7b).

In general, all the figures show positive feedback by mothers and their partners in both usability and technology acceptance, although mothers show higher ratings, as can be observed with their satisfaction: 100% mothers versus 67% partners. Accordingly, there are fewer tasks with less satisfaction for mothers, 33% mothers versus 17% partners (Fig. 7c).

### Discussion and conclusions

There is evidence to suggest that stress during pregnancy causes alterations in the foetus and restricted growth and can result in preterm birth. Proposals supported by mHealth systems that usually monitor pregnancy have the following drawbacks: they only provide information about one health aspect (e.g. nutrition, depression or diabetes); their content is not fully reliable and is only intended to be used by women; the content cannot be adapted to the user’s needs; and no proposal specifically addresses therapeutic interventions in parents at risk of having SGA or premature babies. Moreover, very few studies follow design and usability guidelines to guarantee end-user acceptance and adoption of such systems and their effective use.

This paper presents an approach based on the development of an interactive, adaptive mHealth system intended to assist parents with SGA foetuses to engage on tasks through a psycho-educational intervention programme. To this end, we have also adopted technological methods and tools that encompass stages in a software development life cycle. It is also in line with previous research into intervention programmes for pregnancy, which do not, however, focus on cases with SGA babies. An additional contribution of ours has been to implement certain design decisions in order to equip the mHealth system with certain properties such as adaptability, usability and security. Our proposal also follows Thomas’ recommendation [21] regarding the inclusion of parents not only in the program but also in the system model and prototype, unlike other related work. For the sake of a more comprehensive presentation of the proposal, the system model embraces abstract entities (parent, mother, pregnancy partner, baby, couple and healthcare professionals) in the problem domain (and the relationships between these), and also specific technological elements (author tool, app, database and adaptation engine) present in the solution domain, i.e. the designed mHealth system. Behind these technological elements, we have actually designed other more specific, separate models which are not described in this paper because we consider more technical details to be more suitable for a software type article.

The structure of the technological solution is also flexible thanks to its main components which include the *VivEmbarazo* app, the *AdaptEn* module and the author tool. The parents access structured and updated programme information and tasks through the *VivEmbarazo* app as a frontend of the mHealth system. Its design follows current guidelines and seeks to maximise usability. The *AdaptEn* module reduces development and maintainability time and cost in combination with the author tool used by the therapists, which enables the intervention programme to be built and configured according to user needs.

We have also conducted a proof of concept with the participation of 24 couples. A systematic review and meta-analysis of the data collected from the participants confirms that stress disorder is a risk factor for adverse birth weight and gestational age outcomes [48]. Data about the use of the app have also been analysed and this reveals that the stress of parents who used the *VivEmbarazo* app decreased significantly compared to others who did not use it. This contributes to good practices for improving the emotional health of pregnant women which in turn improves child outcomes [49]. In this regard, following the line of prenatal education for parents using mHealth systems in accordance with other authors such as Kim *et al.* [50], our intention is to reduce the effects of stress on offspring through the mHealth-supported programme to improve parents’ emotional health. Consequently, we found that the babies (and even SGA babies) of the couples following the mHealth-supported programme had a greater weight than the babies of the couples under usual care. Although there may be other influential factors on birth weight, exposure to stress has been shown to increase preterm births and SGA babies [51]. Stress exposure can lead to dysregulation of the hypothalamic-pituitary-adrenal (HPA) axis and cause cortisol release in pregnant women. This mechanism jeopardizes anthropometric fetal development and can result in adverse birth outcomes [49, 52, 53]. One goal of *VivEmbarazo* is help parents to improve emotional health to prevent negative consequences in babies.

In terms of user acceptance and usability during the proof of concept, the participants completed a number of specific questionnaires based on SUS, PSSUQ, eHealth-ITUEM and TAM. The results of the data analysis confirm the usability of the *VivEmbarazo* app and the end-users’ satisfaction with the intervention programme. Although both the mothers and their partners were similarly enthusiastic about the app, the mothers were generally more positive.

The promising results rival more traditional and other more recent technology-based approaches, and warrant and require future research due to the limitations mentioned below. In the future, more data will be obtained and included in order to extend this research work.

We are currently planning to extend the psycho-educational intervention programme to the post-partum phase as requested by healthcare professionals who recognise that most mothers, partners and new-borns need additional assistance. If the programme were to be continued for a year after birth, this new data would be added to the system model.

Another factor to be considered is that during the post-partum phase, different tasks and content must be provided in the intervention programme for the mothers and partners in view of specific conditions, such as, for example, a baby requiring hospitalisation for special healthcare and monitoring. We have equipped the mHealth system with built-in adaptation mechanisms which are capable of extending or creating specific programmes for the post-partum phase. The author tool and the *AdaptEn* module will allow healthcare professionals to create new task models with specific content for such programmes and these should also be validated in the real clinical setting.

Limitations: This is a preliminary study and as such has limitations, particularly with regard to the sample size. However, the promising results obtained will encourage us to continue applying the mHealth programme to a larger sample. Another limitation is the lower participation rate of partners and this can affect some of the results obtained in terms of usability and acceptance. It would, therefore, be advisable to implement involvement strategies for partners and to include more specific, relevant content for them in the mHealth programme.

## Supplementary Information

**Additional file 1.** This file contains the questionnaires developedand used for this intervention program, which were adapted from SUS,PSSUQ and TAM questionnaires [26, 28, 23]. Title of data:Questionnaires. Description of data: The questionnaires areassociated with each task so that parents can assess their learningprogress on general topics (healthcare, medical advice, foetusstimulation and emotion management) included on thepsycho-educational intervention programme.

## Data Availability

The software and datasets generated and/or analysed will be available after the development of the next prototype version and use.

## Abbreviations

mHealth: Mobile health
app: Mobile software application
SGA: Small for Gestational Age
ICT: Information and Communication Technologies
ANC: Antenatal care
PSS: Perceived Stress Scale
EPDS: Edinburgh Postnatal Depression Scale
UI: User Interface
HTTP: Hypertext Transfer Protocol
