# Supplementary material for: A psycho-educational intervention programme for parents with SGA foetuses supported by an adaptive mHealth system: design, proof of concept and usability assessment
Source: BMC Med Inform Decis Mak. 2022 Nov 11;22(Suppl 4):291. doi: 10.1186/s12911-022-02036-9 (PMC9650852; doi:10.1186/s12911-022-02036-9)
Supplement: Supplementary file 1 — Additional file 1. This file contains the questionnaires developedand used for this intervention program, which were adapted from SUS,PSSUQ and TAM questionnaires [26, 28, 23]. Title of data:Questionnaires. Description of data: The questionnaires areassociated with each task so that parents can assess their learningprogress on general topics (healthcare, medical advice, foetusstimulation and emotion management) included on thepsycho-educational intervention programme. [file 12911_2022_2036_MOESM1_ESM.pdf]

Supplementary material

Sara Balderas-Díaz[1], María José Rodríguez-Fórtiz[2], José Luis Garrido[2], Mercedes Bellido-González[3], and Gabriel Guerrero-Contreras[1]

[1]*Department of Computer Science and Engineering, University of Cádiz, Cádiz, Spain*
[2]*Software Engineering Department, University of Granada, Granada, Spain*
[3]*Biosanitary Research Institute (Ibs.Granada), Granada, Spain*

Questionnaires

As we outlined in this research work and discussed in the proof of concept subsection, various questionnaires are associated with each task so that parents can assess their learning progress on general topics (healthcare, medical advice, foetus stimulation and emotion management) included on the psycho-educational intervention programme. So as not to overwhelm the parents at the end of the intervention programme with specific questionnaires about usability and acceptance, such questions were included on some of the questionnaires together with other general topic questions. Due to the large number of questions, and for reasons of clarity, we only show questions which assess usability and acceptance in the following two tables. The questionnaires also assessed other Health-ITUEM concepts and these results are shown in Table 1, which includes error prevention in notification and performance speed in functionality, and in Table 2, which presents questions about completeness and required information in perceived usefulness. Perceived ease of use includes questions about memorability, learnability and competency. There are no questions about flexibility/customizability since only professionals are offered the change or adaptation option for designing different intervention programmes.

Table 1: **Questionnaire of Usability** (each question corresponds to task number and number of questions in the designed intervention programme).

User Interface Design	Do you think that the colours used in the app are appropriate? Score it from 1 to 5 (1 = Not appropriate, 5 = Very appropriate)
	Do you think that the fonts used in the app are appropriate? Score it from 1 to 5 (1 = Not appropriate, 5 = Very appropriate)
	Can you identify the buttons to be pushed? (No; I have problems identifying them; Yes)
	Do you know what tasks are completed and what tasks are not? (No; I have doubts; Yes)
	Is the language used in the "tasks" appropriate? Score it from 1 to 5 (1 = Not appropriate, 5 = Very appropriate)
Navigation	Do you think it is appropriate that the content of a "task" is structured in sections such as "explanation", "activities" and "To learn more"? (1 = Not appropriate, 5 = Very appropriate)
	Is it easy for you to know if you are accessing a list of tasks, a specific task, or carrying out an activity or questionnaire? (No; Sometimes I doubt; Yes)
Notification	Has the app shown you an error message or shut down unexpectedly? Score it from 1 to 5 (1 = Yes, more than five times, 5 = Never)
Functionality	When you are watching a video, do you have enough time to pay attention to what it says and to read the messages which appear in it? (No; No, I have only partially read the content; Yes)
	Have you ever been unable to view a video or open a link to "learn more"? (Yes, I have had a problem; I am not sure; I have never had problems with this)

Table 2: **Questionnaire of Technology Acceptance (Perception of Utility, Ease of use, Attitude towards the actual use, and Satisfaction).**

Perceived usefulness	Music. Do you think that this activity is useful to communicate with your baby? Score it from 1 to 5 (1 = Not at all, 5 = Very much)
	Physical exercise. Score how this task has been useful to you. (1 = Not at all, 5 = Very much)
	Nutrition. Score how this task has been useful to you. (1 = Not at all, 5 = Very much)
	Hygiene and care. Check to what extent are you satisfied with the information received in this task? Score it from 1 to 5 (1 = Not at all, 5 = Very much)
	Light. How do you appreciate the utility of the light activities to the development of the baby? Score it from 1 to 5 (1 = Not at all, 5 = Very much)
	Do you think that the topics presented in the different "tasks" of this app are appropriate? Score it from 1 to 5 (1 = Not appropriate, 5 = Very appropriate)
	Do you think that the number of tasks is appropriate? Score it from 1 to 5 (1 = Not appropriate, 5 = Very appropriate)
	Is the information provided in the "learn more" section interesting for you? Score it from 1 to 5 (1 = Not interesting, 5 = Very interesting)
	Has the app VivEmbarazo been useful to you? Score it from 1 to 5 (1 = It has not been useful, 5 = It has been very useful)
Perceived ease of use	Do you think that this app (VivEmbarazo) is intuitive and easy to use? Score it from 1 to 5 (1 = Not at all, 5 = Very intuitive)
	If you want to search for specific content of interest to you, do you think that it could be difficult to you to find it? Score it from 1 to 5 (1 = Very difficult, 5 = No difficult at all)
	Do you need the help of another person to be able to use the app? Score it from 1 to 5 (1 = A lot of help, 5 = No help)
	Which of the "Tasks" have been especially complex to you? (Choose one or more from 1 to 20)
Attitude toward actual use	Have you ever used an app to follow the pregnancy, apart from this? (No; Yes, one time; Yes, several times)
	Positive emotion. Do you feel good after carrying out these activities? Score your mood from 1 to 5 (1 = Not good, 5 = very good)
	Taste. Did you feel good while carrying out the taste activity? Score it from 1 to 5 (1 = Not at all, 5 = Very much)
	Do you think that the duration of the activities in this app is appropriate? Score it from 1 to 5 (1 = Not appropriate, 5 = Very appropriate)
	Are you comfortable using the app? Score it from 1 to 5 (1 = Not comfortable at all, 5 = Very comfortable)
Perceived satisfaction	Nutrition advice. Check to what extent are you satisfied with the information received in this task? Score it from 1 to 5 (1 = Not at all, 5 = Very much)
	Which of the next "Tasks" do you like more? (Choose one or more from 1 to 20)
	Which of the next "Tasks" do you like less? (Choose one or more from 1 to 20)
